# Historical Use of Cultivars as Parents in Florida and Louisiana Sugarcane Breeding Programs

**DOI:** 10.1155/2015/257417

**Published:** 2015-01-20

**Authors:** James Todd, Barry Glaz, David Burner, Collins Kimbeng

**Affiliations:** ^1^Everglades Research & Education Center, 3200 E. Palm Beach Road, Belle Glade, FL 33430-4702, USA; ^2^USDA-ARS Sugarcane Research Unit, Houma, LA 70360, USA; ^3^Louisiana State University AgCenter Sugar Research Station, St. Gabriel, LA 70776, USA

## Abstract

Sugarcane (*Saccharum* L. spp. hybrids) growers depend on breeding programs for new, high-yielding cultivars that have resistance to abiotic and biotic stresses, so breeders continually seek out widely adapted, high yielding germplasm to be used as parents for their programs. Cultivars are sometimes used for this purpose, but their use may be minimized to prevent genetic diversity erosion. The purpose of this study was to determine the importance of cultivars as parents in three USA (one in Florida and two in Louisiana) sugarcane breeding programs by quantifying the percentage of cultivars that had these parental groupings based on published registrations and crossing records. The percentage of cultivars with at least one commercial parent for each program was 81.8%, 77.5%, and 64.3% for the Houma (Ho), Louisiana, Canal Point (CP), Florida and Louisiana State University (LSU) programs, respectively, but cultivars were recently used as parents in only 11.8% (Ho), 16.39% (CP), and 34.3% (LSU) of crosses. The results indicate that the CP and Ho programs should consider increasing the use of cultivars as parents in their breeding programs to increase the probability of selecting potential commercial genotypes, but this should be balanced with high diversity crosses to avoid the loss of diversity.

## 1. Introduction

Mainland US sugarcane breeding programs employ recurrent selection principles that continually identify the highest yielding germplasm with acceptable stress resistance and make the majority of crosses in their breeding programs using these genotypes as parents. Throughout this paper, sugarcane genotype is used to denote a genetically unique individual derived from sexual recombination. Genotypes are most often tested in different stages of selection programs and are generally named in an early stage of selection. Later, once more of the data are available for a given genotype, some are used as parents in crosses. Cultivar denotes a genotype that has been publicly released for commercial use. We use the term commercial parent to describe a cultivar that is used as a parent in a cross.

Three breeding programs were researched in this study. The two USDA-ARS breeding programs are based in Houma, LA, Canal Point, FL, and the third Louisiana State University program in St. Gabriel, LA. Each breeding program has different breeding strategies that address the different regions they serve. Florida has a subtropical climate where cold tolerance is a concern, although damaging freezes do not occur every year. However, Louisiana has a colder climate with regular damaging freezes, and cold tolerance is more important in Louisiana than in Florida. The Louisiana sugarcane industry is comprised of a relatively large number of growers with smaller farms while the Florida industry has large corporations and generally much greater land areas per farm owner than in Louisiana. For example, a representative farm size for the Louisiana industry would be 404.7 ha [1] while a representative farm size in Florida would be 2000 ha [2]. Such industry characteristics result in programmatic differences in the number and type of crosses made, type of plants selected, and numbers of cultivars released.

Houma and LSU programs, whose origin can be traced to 1885 [3], are highly collaborative and mutually strive to improve productivity of the Louisiana sugarcane industry [4]. Both Louisiana programs conduct sugarcane breeding and selection activities [5]. Crossing through early stage clonal testing and selection are conducted independently by each of the two institutions, and midway through clonal selection stages promising genotypes from the two are jointly evaluated by the three Louisiana organizations. The American Sugar Cane League (ASCL) assists with the final multilocation testing stage and has primary responsibility for seed increase and distribution to growers upon varietal release. In accordance with the three-way agreement, all three Louisiana organizations jointly decide on a cultivar release [5].

Sugarcane crosses have been made in Louisiana since 1948 [6] by Louisiana State University. The current Louisiana breeding program is conducted by Louisiana State University Agricultural Center (LSU) at St. Gabriel, LA, along with the USDA-ARS Sugarcane Research Unit at Houma, LA, in cooperation with the ASCL located at Thibodaux, LA, under a three-way cooperative agreement [7]. The ASCL is Louisiana's sugarcane grower and processor organization and contributes professional expertise and financial support [7]. The Houma program emphasizes germplasm enhancement through basic breeding [8]. Basic breeding is a scheme to intercross the desirable alleles from diverse wild germplasm into a composite breeding material that will be used to backcross into elite cultivars [9]. The Florida and Louisiana programs have long been closely affiliated. Prior to 1972, basic- and commercial-type sugarcane crosses for the Houma program were made only at the USDA-ARS Research Station at Canal Point (CP), Florida. Since then crossing efforts specifically targeting germplasm enhancement were initiated at Houma [8]. Crossing facilities at Houma are primarily for basic-type crosses, while CP serves as the main source of Houma's commercial-type crosses using Louisiana germplasm.

Houma cultivars from CP 65-357 to CP 79-318 received the CP name designation even though germplasm had been selected at Houma. This included the BC 1 cultivar TucCP 77-42 which was selected in Argentina from a cross made at Houma [10]. Cultivars released from the LSU program have an “L” name designation while those from the Houma program have an Ho name designation. A compound naming designation was developed to assure that a cultivar name appropriately reflected the breeding groups involved in its development [9]. For example, HoCP 85-845 indicates that the cross was made at CP and the clone was selected at Houma [11].

Sugarcane breeding in the United States for the Louisiana breeding program began in Canal Point Florida in 1919 [12] but from 1960 breeding efforts were also directed for Florida region [13]. The current Canal Point program consists of a three-way cooperative agreement between the USDA-ARS Research Field Station at Canal Point (CP), the University of Florida Everglades Research and Education Center located at Belle Glade, and the Florida Sugarcane League located at Clewiston, Florida. Cultivars are recommended for release by the vote of a committee with representatives from each of these three organizations and carry a CP name designation.

Florida and Louisiana programs also share germplasm between themselves resulting in combined LCP, HoCP, and LHo name designations.

Deren [14] traced the genealogy of US sugarcane germplasm and found that 10 clones were the source of 90% of Florida germplasm and that two ancestors were the source of 90% of Louisiana germplasm. He noted that the diversity of sugarcane was buffered due to multiple interspecific crosses in the respective breeding programs. These include wild species and early generation intergeneric and interspecific crosses used to broaden genetic diversity and increase disease resistance. This is exemplified by the Houma, LA, breeding program which maintains two general types of germplasm: basic germplasm, typically F1 to BC2 generations, and elite or nearly elite commercial germplasm (typically BC3 and higher generations) [5]. There are also basic crosses made in Canal Point which are also evaluated for cold tolerance and in the energycane program, but the main focus of the Canal Point program is sugarcane cultivar development. In most crops, including sugarcane, new cultivars are selected from the progeny of crosses of commonly grown cultivars and other elite materials [5]. There is some evidence of past successful use of cultivars as parents in other programs including POJ2878 which was the parent of 181 sugarcane varieties worldwide and Co419 that was the parent of 81 sugarcane cultivars including two of China's most widely grown cultivars [15, 16]. Other popular cultivars in China include ROC22 which has been used as a parent in crosses since 2002 and at least five of its progeny have been released as cultivars including Guitang 04-1001, Guitang 29, [17], Dezhe 03-83 [18], and Liucheng 03-182 [16]. Cultivars are sometimes used in crosses, but their importance as breeding parents in sugarcane breeding programs has not been documented. The purpose of this study was to determine the importance of cultivars as parents in Florida and Louisiana public sugarcane breeding programs by quantifying the percentage of cultivars derived from crosses with commercial parents.

## 2. Materials and Methods

Three public sugarcane breeding and selection programs were included in the study: CP, Houma, and LSU. Records of cultivar pedigrees were collected using existing pedigree dendrograms and published cultivar registrations. Information for some cultivars was obtained from [19, 20]. The search was limited to cultivars grown for sugar and biofuel. Genealogies for the Florida program began with cultivar CP 63-306 which was released in 1971. Earlier cultivars in the CP program are not evaluated, but they are noted as parents thereby covering 52 years of breeding. Some CP cultivars developed by the Houma breeding program, before the compound naming system, were often planted in Florida. Early CP cultivars selected for release to the Louisiana industry were counted as Houma cultivars and those selected for release in Florida were counted as CP cultivars.

Cultivars developed by LSU in this study began with L 62-96 which was released in 1969.

Crossing records from CP for 2000–2006 and 2010–2012 and LSU for 2003–2012 and Houma from 1992 to 2012 were obtained from crossing records of each program to compare the number of cultivars that was used as parents of other cultivars and the use of cultivars as parents in the total number of crosses generated. Within the CP records all crosses are recorded including those made for CP and Houma and the crosses made for both. The years 2007–2009 were not included in CP counts because the difference between crosses used in Florida and crosses used for Houma, Louisiana was not recorded and the crosses for Florida and Louisiana could not be distinguished. Ten years were assumed to be sufficient for CP program based on the large number of crosses (6945 total crosses) included in the study. Additional years were included for the Houma program (6630 total crosses) to approximate the number crosses of the CP program. The number of crosses for LSU was restricted to 4423 total crosses as additional crossing records for this program were not available.

To calculate the percentage of cultivars with at least one commercial parent, the presence of one or two commercial parents was counted as one per cultivar progeny then divided by the total number of cultivar progeny (at least one parent cross/total number of crosses ∗ 100). The percentage of all commercial parents was calculated by adding all the commercial parents and dividing them by the total number of parents, including those with unknown parents. The percentage of cultivars calculated from crossing records was calculated by dividing the total number of times cultivar parents were used in the cross by the total number of times all parents were used and the selfs were treated as one parent use (cultivar parents/all parents ∗ 100). Two types of Chi-square tests were conducted using SAS PROC Freq: a test between the breeding locations and a proportion test of cultivar versus noncultivar within breeding locations (*P* < 0.05).

## 3. Results and Discussion

There were 71 cultivars from the CP program reviewed (Table 1). Of these 71 cultivars 77.5% of them had at least one cultivar parent, and 54.6% of the total parents were cultivars (Table 2). There were 42 cultivars used as parents in the crosses that developed these 71 cultivars, and each of these cultivars was a parent of another cultivar at an average of 1.83 times (sum of the times a particular cultivar was used as parent divided by the total number of cultivar parents). Cultivars were used as female parents of cultivars 47 times and as male parents 30 times including two selfs; CP 89-2143 [21] was the parent of CP 00-1101 and HoCP 91-552 [22] which was the parent of CP 00-2180 [23]. The most successful commercial parent was CP 68-1067 [24], which was used as a parent six times from which new cultivars were selected. The female and male parents of CP 68-1067 are CP 52-68 [20] and CP 57-603 [25], respectively, and they were also cultivars. Cultivar progeny of CP 68-1067 are CP 75-1082 [26], CP 75-1632 [27], CP 77-1776 [28], CP 78-1628 [29], CP 80-1743 [30], and CP 81-1384 [31]. Out of six cultivars five were produced when CP 68-1067 was used as a female parent and one (CP 80-1743) when used as male parent. CP 80-1743 was the most widely grown sugarcane cultivar in Florida for several years and CP 78-1628 was the most widely grown sugarcane cultivar on sand soils in Florida for several years [32]. Most of the cultivar progeny of CP 68-1067 had high or moderate commercial recoverable sucrose, except CP 75-1082 which had high cane tonnage and low commercial recoverable sucrose. Some of the cultivar progeny of CP 68-1067 also had cultivar grand-progeny. CP 75-1082 was the female parent of CP 86-1633 [33] and CP 80-1743 was the female parent of CP 88-1762 [34], CP 97-1944 [35], and CPCL 02-0926 [36]. CP 88-1762 became the most widely grown cultivar in Florida in 2010 [37].

If one could rate the breeding success of a cultivar by having the most progeny that become cultivars, then the second most successful cultivar parent in the CP program was CP 70-1133 [38], which had five of its progeny that became cultivars (Table 1). CP 56-63 [39], which was the female parent of a polycross that created CP 70-1133, was also a cultivar. The cultivar progeny of CP 70-1133 were CP 75-1082, CP 75-1632, CP 80-1827 [40], CP 82-1592 [41], and CP 84-1198 [42]. CP 80-1827 was the most widely grown sugarcane cultivar in Florida from 1995 to 1998 [43]. There are also cultivar grand-progeny of CP 70-1133: CP 75-1082 was the female parent of CP 86-1633; CP 80-1827 was the female parent of CP 89-1509 [44] and CP 92-1641 [45]; CP 82-1592 was the female parent of CP 92-1666 [46]; and CP 84-1198 was the male parent of CP 96-1252 [47], CP 04-1844 [48], and CPCL 99-4455 [49].

The parents of 22 Houma cultivars were reviewed (Table 3). Of these 22 cultivars 81.8% had at least one cultivar parent, and 56.82% of the total parents were cultivars (Table 3). The most successful cross in the Houma program was between CP 65-357 [50] and L 65-69 [51]. The hugely successful Louisiana cultivar, CP 65-357, was grown on more land area (71%) than the sum of all other cultivars in 1980 [52]. It took two decades before another cultivar, LCP 85-384, assumed an equally prominent lead above all other cultivars, with 91% of the acreage in 2004 [53]. The cross of cultivars CP 65-357 × L 65-69 produced four cultivars: CP 73-351 [54], CP-74-383 [55], CP 76-331 [56], and CP 79-318 [57]. None of the cultivars from the L 65-69 × CP 65-357 crosses parented other cultivars. CP 65-357 was not a parent of any LSU cultivar (Table 4). More cultivars and parents were shared between the two Louisiana programs (Tables 3 and 4) than between Louisiana and Florida programs (Table 1), probably reflecting the different cultivar specific requirements of their respective industries.

The parents of 14 LSU cultivars were reviewed and of these 64.3% had at least one cultivar parent, and 42.86% of the total parents were cultivars. Of these, three cultivars were repeated as parents. CP 52-68 [20] was a parent of three commercial progenies. CP 48-103 [20] had two commercial progenies and LCP 85-384 [58] had three commercial progenies (Table 4).

It is likely that these programs actually have higher percentages of cultivars with commercial parents. For example, if we evaluate the parents in the CP program that were not identified as cultivars, we find that there were six unknown genotypes because of labeling issues and 12 unknown males because they were in polycrosses. Thus, there is a chance that some of these 18 unknown parents were cultivars. The paternity of these polycrosses could be determined with further study using molecular markers [59].

A total of 46 genotypes that were parents of commercial progeny in the CP program were noncommercial mostly elite germplasm. The most successful of these parents was CP 84-1322; it was the male parent of three cultivars. For two of three cultivars the female parent was also a cultivar (crosses that developed CP 92-1641 and CP 92-1666). Also, the parents of CP 84-1322 were both commercial cultivars (CP 52-068 [20] and CP 72-2086 [60]). CP 72-2086 was the most widely grown cultivar in Florida in 1994 [43]. Most noncommercial germplasm only parented commercial progeny one or two times. The Houma program had two noncommercial elite parents producing commercial progeny two times each CP 83-644 and CP 61-39, and the LSU program also had two noncultivar elite parents producing commercial progeny two times each CP 77-310 and L 93-365.

Four cultivars from Houma were derived from basic parental clones (Table 3) including SES 234 (*S. spontaneum* L.) and three US clones. Houma clones selected in basic breeding were previously assigned US [United States] breeding numbers, but basic selections are now given Ho designations. Basic clones typically were used in the breeding program as nonrecurrent parents for backcrossing with selected interspecific hybrids used as recurrent parents [10], and cultivars were often the recurrent parent. In practice, crosses usually were constructed taking into consideration the parental pedigrees to minimize potential effects of inbreeding. For example, LCP 85-384 [58] has occurred frequently as a parent in the Louisiana programs (Tables 3 and 4), so caution is needed to minimize full- and half-sib crosses with this cultivar. The high number of noncommercial crosses in the Houma program (Table 2) indicates that the basic program is strong. But since the released cultivars have ancestry which is primarily commercial, new methods should be sought to incorporate basic germplasm in such a way to develop successful cultivars that utilize better the diversity of the basic program.

The crossing records at CP were gathered from recent records to see how the programs have acted recently and see how they might change. Programs differed significantly in the percentage of cultivars used in crosses in the order LSU > CP > Houma, 34.3, 16.4, and 11.8%, respectively (Table 2). LSU had nearly a 2- to 3-fold greater use of cultivars as parents than the other programs. Overuse of cultivars in crosses could cause a reduction in genetic diversity.

Of the 71 CP program cultivars surveyed, 55 (77.8%) had at least one commercial parent. Over half of the parents (77/141∗100 = 54.6%) of cultivars in the CP program were cultivars. According to the crossing records examined, cultivars were used in only 16.4% of the crosses in this program (Table 2). Houma used cultivars as parents in significantly (Chi-square *P* < 0.05) fewer crosses (11.8%) but had a larger percentage of parent cultivars (56.8%) and cultivars with at least one parent (81.8%) but these differences were not significant. But the proportions of cultivars with at least one cultivar parent to those with no cultivar parentage in the CP (77.46) and Houma (81.82) programs were significantly unequal. The proportion of at least one cultivar parent in the LSU (64.29) program was not significantly unequal indicating that the CP and Houma programs have released proportionally more cultivars with commercial parents. The LSU breeding program used cultivars in a greater percentage of their crosses (34.3%) than CP and Houma. However, there were fewer cultivars with parent cultivars (42.9%) and few cultivars with at least one cultivar parent (64.3) compared to the CP or Houma programs. The crossing percentages from the different programs do not directly relate to the percentages of cultivars with cultivar parents because the crossing period examined does not correspond with the time periods when most of the older cultivars were developed. The current crossing records do indicate that commercial cultivar crossing is not a priority and could be increased. The difference between programs for total commercial parents and cultivars, at least one cultivar parent, was not significantly different between the breeding programs, but the percentage of cultivars used in crossing was significantly different between programs. Our results indicate that cultivars are an excellent source of successful parents in all three programs and even though the programs have different breeding strategies, the cultivars they release are not significantly different in their proportions of total cultivar parents. LSU uses significantly more cultivars in crosses, but the proportion of at least one cultivar parent is proportional to noncultivars unlike the other programs (Table 2). The Houma program had percentages similar to CP even though it differs somewhat from the other two programs due to its emphasis on germplasm enhancement through basic breeding [5], with lesser importance placed on commercial crosses. Basic and commercial germplasm gradually merge through recurrent selection to the point at which cultivars are released for sugar or bioenergy. Cultivars also were used in 16.4% (CP), 11.8% (Houma), and 34.3% (LSU) of total crosses demonstrating their importance for contributing superior genes in the breeding pool at each location. The lower frequency of cultivars in total crosses at Houma probably reflects the greater use of basic germplasm in this program compared to that at LSU. But the genotype crosses at CP include mostly elite germplasm crosses and a few basic and energycane crosses. Energycane breeding which represented 13% of the CP crosses (159 crosses) in 2012 overlaps somewhat with basic breeding at CP and the industry for energycane is relatively new so not many cultivars have been released. There is also proportion of basic crosses shared between Houma and CP programs.

The choice of germplasm for breeding is biased in subtropical climates by the problem of unreliable flowering [61]. Often promising germplasm or cultivars are not selected for crossing because they will not flower under the conditions at the breeding station. Some promising foreign germplasm at Canal Point is not selected for crossing because they will not flower (personal communication). This could explain why there is less exotic material in the pedigrees of cultivars. Also lack of flowering synchrony will limit the types of crosses that can be made [61]. This could have skewed the use of cultivars in crossing at each breeding program.

If breeding programs increase the percentage of cultivars in their crossing programs, it should be done strategically to ensure that robust sources of diversity are maintained. Loss of diversity could hamper long-term breeding progress. Broadness of germplasm among the parental pool is an important measure of genetic diversity [62], and restricting parents to cultivars would narrow the parental gene pool. Maintaining a diverse germplasm and having the flexibility to import new germplasm are also important because of the constant need to incorporate new disease resistance genes into the parental pool, but the selection of these for crossing could be affected by the ability of these parents to flower. A possible reason why cultivars with cultivar parents are chosen is adaptability. The short cold Louisiana season and the muck organic soils in Florida are unique environments that probably favor particular gene combinations within existing cultivars. A future challenge will be to find efficient ways to quickly incorporate diversity and disease resistance into high performing germplasm like commercial cultivars.

There is a group of cultivars (Table 5) that did not produce commercial progeny. This could be due to not being crossed or deficiencies in their progeny. This should be investigated in the future to clarify better what useful germplasm is. It could be that the yield of some commercial and elite germplasm is due to heterosis and nonadditive genes. As molecular breeding technology becomes more practical and economically feasible it would be interesting to locate quantitative trait loci (QTL) of good parents then trace them within a lineage and compare them to cultivars that did not have successful progeny. This would allow us to understand better how QTLs from diverse backgrounds interact [63].

## 4. Conclusions

Sugarcane cultivars have been found to be acting as successful parents in all three breeding programs. A large percentage of the total number of cultivars in all programs has at least one commercial parent. In the LSU sugarcane breeding program the proportion of commercial parents to noncommercial parents was not significantly different but in the more recent crossing time period analyzed cultivars were crossed significantly more frequently than in the other programs.

The Canal Point and Houma programs did have significantly higher proportion of their cultivars with at least one commercial parent. Programmatic differences reflect, to some extent, philosophical approaches and responses to their respective industries. For example, the Florida program is larger than the two Louisiana programs combined, serves a subtropical climate with a longer growing season, and releases more cultivars to larger growers. Conversely, the Louisiana industry serves many growers with smaller farming operations with a shorter growing season who prefer that cultivars demonstrate early maturity prior to release.

## Figures and Tables

**Table 1:** Cultivars and their parents developed by the breeding and selection program at the USDA-ARS Sugarcane Field Station in Canal Point, FL.

Cultivar	Female parent	Male parent	Cultivar	Female parent	Male parent
CP 63-306	CP 50-28^a^	CP 52-15	CP 88-1508	CP 81-1238^a^	CP 78-1610
CP 63-588	Cl 54-1910^a^	CP 57-120	CP 88-1540	CP 78-1610	CP 81-1238^a^
CP 68-1026	Cl 47-83	CP 57-614^a^	CP 88-1762	CP 80-1743^a^	85 P6^b^
CP 68-1067	CP 52-68^a^	CP 57-603^a^	CP 88-1834	CP 72-1210^a^	LCP 81-30
CP 69-1052	CP 56-59^a^	CP 62-374^a^	CP 89-1509	CP 80-1827^a^	Polycross^b^
CP 70-1133	CP 56-63^a^	67 P6^b^	CP 89-2143	CP 81-1254^a^	CP 72-2086^a^
CP 70-1527	CP 62-374^a^	CP 57-526	CP 89-2376	Unknown	Unknown
CP 71-1038	CP 52-68^a^	CP 56-59^a^	CP 89-2377	Unknown	Unknown
CP 72-1210	CP 65-357^a^	CP 56-63^a^	CP 92-1213	CL 73-0239^a^	CP 85-1498
CP 72-2086	CP 62-374^a^	CP 63-588^a^	CP 92-1641	CP 80-1827^a^	CP 84-1322
CP 73-1547	CP 66-1043	CP 56-63^a^	CP 92-1666	CP 82-1592^a^	CP 84-1322
CP 74-2005	CP 66-1043	CP 63-588^a^	CP 94-1100	CP 81-1238^a^	CP 88-2045
CP 75-1082	CP 68-1067^a^	CP 70-1133^a^	CP 94-1340	CP 87-1733	CP 86-1665
CP 75-1553	CP 68-1026^a^	73 P2^b^	CP 96-1252	CP 90-1533	CP 84-1198^a^
CP 75-1632	CP 68-1067^a^	CP 70-1133^a^	CP 96-1602	CP 81-1425	Polycross^b^
CP 77-1776	CP 68-1067^a^	CP 68-1022	CP 97-1944	CP 80-1743^a^	Polycross^b^
CP 78-1247	CP 68-1067^a^	CP 57-614^a^	CP 97-1989	CP 75-1091	CL 61-620^a^
CP 78-1628	CP 65-357^a^	CP 68-1026^a^	CP 98-1029	CP 91-1980	CP 94-1952
CP 78-2114	Unknown	Unknown	CP 00-1101	CP 89-2143^a^	CP 89-2143^a^
CP 80-1557	CP 71-1194	Polycross^b^	CP 00-1446	CP 93-1607	CP 91-1150
CP 80-1743	CP 72-2083^a^	CP 68-1067^a^	CP 00-2180	HoCP 91-552^a^	HoCP 91-552^a^
CP 80-1827	CP 70-1133^a^	CP 73-1311	CP 01-1372	CP 94-1200	CP 89-2143^a^
CP 81-1238	CP 71-1027	Polycross^b^	CP 04-1566	CP 89-2377^a^	CP 96-1252^a^
CP 81-1254	CP 72-1210^a^	Polycross^b^	CP 04-1844	CP 97-1989^a^	CP 84-1198^a^
CP 81-1302	CP 72-2079	CP 71-1086	CP 04-1935	CP 94-2059	CP 84-1322
CP 81-1384	CP 68-1067^a^	CP 74-2013	CP 05-1526	CP 98-1029^a^	CP 88-1162
CP 82-1172	CP 75-1091	CP 75-1283	CPCL 97-2730	CL 75-0853^a^	CL 88-4730^a^
CP 82-1592	CP 72-1210^a^	CP 70-1133^a^	CPCL 99-4455	CL 90–4643	CP 84-1198^a^
CP 84-1198	CP 70-1133^a^	CP 72-2086^a^	CPCL 00-4111	CL 83-3431^a^	CL 89-5189^a^
CP 84-1591	CP 72-1370	CP 68-1022	CPCL 02-0926	CP 80-1743^a^	CL 92-0046
CP 85-1308	R 567	CP 74-2013	CPCL 02-1295	CP 88-1762^a^	CL 91-1637
CP 85-1382	CP 74-2005^a^	82 P 14^b^	CPCL 02-6848	CL 92-2533	Poly 01-9^b^
CP 85-1432	CP 70-1527^a^	Polycross^b^	CPCL 05-1102	CL 89-5189^a^	CL-88-4730^a^
CP 85-1491	CP 75-1553^a^	CP 72-2086^a^	CPCL 05-1201	CL 87-2882	CL 93-2679
CP 86-1633	CP 75-1082^a^	CP 78-1140	CPCL 05-1791	CP 96-1252^a^	CL 90-4725^a^
CP 88-1165	CL 61-620^a^	CP 81-1302^a^			

^a^Indication that clone was a cultivar used as a parent.

^b^Male polycross (pollen mix).

**Table 2:** Percentage of commercial parentage of cultivars, cultivars with at least one cultivar parent, percentage of cultivars used in crosses, and total crosses for breeding programs at Canal Point (CP), Houma, and Louisiana State University (LSU).

	Cultivars	At least one cultivar	Commercial parents in crosses^a^	Total crosses
%				(no.)
CP	54.61	77.46^*^	16.39^∗‡^	6945
Houma	56.82	81.82^*^	11.77^∗‡^	6630
LSU	42.86	64.29	34.29^∗‡^	4423

^a^For CP crosses are from 2000 to 2006 and 2010 to 2012, for LSU crosses are from 2003 to 2012, and for Houma crosses are from 1993 to 2012.

^*^Significantly different at 0.05 for the Chi-square test of equal proportions (cultivar versus noncultivar within each program).

^‡^Significantly different at 0.05 for the Chi-square test comparing the percentages of the different breeding programs.

**Table 3:** Cultivars and their parents developed by the breeding and selection program located at the USDA-ARS Sugarcane Research Unit in Houma, LA.

Cultivar	Female parent	Male parent
CP 65-357	CP 52-68^a^	CP 53-17
CP 67-412	CP 44-155^a^	CP 53-16
CP 70-321	CP 61-39	CP 57-614^a^
CP 70-330	CP 61-39	CP 57-614^a^
CP 72-370	CP 61-37^a^	CP 52-68^a^
CP 72-356	CP 63-361	CP 62-258^a^
CP 73-351	CP 65-357^a^	L 65-69^a^
CP 76-331	CP 65-357^a^	L 65-69^a^
CP 79-318	CP 65-357^a^	L 65-69^a^
CP 74-383	CP 65-357^a^	L 65-69^a^
Ho 00-961	US 94-1	HoCP 91-552^a^
Ho 02-113	SES 234	LCP 85-384^a^
Ho 05-961	CP 83-644	TucCP 77-42^a^
Ho 07-613	HoCP 00-905	HoCP 96-540^a^
Ho 95-988	CP 86-941	US 89-12
HoCP 00-950	HoCP 93-750	HoCP 92-676
HoCP 04-838	HoCP 85-845^a^	LCP 85-384^a^
HoCP 85-845	CP 72-370^a^	CP 77-403
HoCP 91-552	LCP 81-10	CP 72-356^a^
HoCP 91-555	CP 83-644	LCP 82-94
HoCP 96-540	LCP 86-454^a^	LCP 85-384^a^
TucCP 77-42	CP 71-321	US 72-19

^a^Indication that clones was a cultivar used as a parent.

Cultivars were released through a three-way agreement between Houma, Louisiana State University, and the American Sugar Cane League.

**Table 4:** Cultivars and their parents developed by the breeding and selection program located at Louisiana State University (LSU) in Baton Rouge, LA.

Cultivar	Female parent	Male parent
L 60-25	CP 52-68^a^	CP 48-103^a^
L 62-96	CP 52-68^a^	CP 44-154^a^
L 65-69	CP 52-1	CP 48-103^a^
LCP 82-89	CP 52-68^a^	CP 72-370^a^
LHo 83-153	CP 77-405	CP 74-339
LCP 85-384	CP 77-310	CP 77-407
L 79-1002	CP 52-68^a^	Tainan
LCP 86-454	CP 77-310	CP 69-380
L 99-226	HoCP 89-846	CP 81-30
L 99-233	CP 79-348	HoCP 91-552^a^
L 03-371	CP 83-644	LCP 82-089
L 97-128	LCP 81-10	LCP 85-384^a^
L 01-299	L 93-365	LCP 85-384^a^
L 01-283	L 93-365	LCP 85-384^a^

^a^Indication that clone was a cultivar used as a parent.

Cultivars were released through a three-way agreement between USDA-ARS Houma, LSU, and the American Sugar Cane League.

**Table 5:** List of cultivars that did not produce cultivar progeny in the time period surveyed.

Co 281	CP 55-30	CP 88-1165	Ho 05-961
Co 290	CP 59-73	CP 88-1508	Ho 07-613
CP 00-1101	CP 63-306	CP 88-1540	Ho 95-988
CP 00-1446	CP 67-412	CP 88-1834	HoCP 00-950
CP 00-2180	CP 70-321	CP 89-1509	HoCP 04-838
CP 01-1372	CP 70-330	CP 89-2376	HoCP 85-845
CP 04-1566	CP 71-1038	CP 92-1213	HoCP 91-555
CP 04-1844	CP 73-1547	CP 92-1641	L 01-283
CP 04-1935	CP 73-351	CP 92-1666	L 01-299
CP 05-1526	CP 74-383	CP 94-1100	L 03-371
CP 28-11	CP 75-1632	CP 94-1340	L 60-25
CP 28-19	CP 76-331	CP 96-1602	L 62-96
CP 29-103	CP 77-1776	CP 97-1944	L 79-1002
CP 29-116	CP 78-1247	CPCL 00-4111	L 97-128
CP 29-120	CP 78-1628	CPCL 02-0926	L 99-226
CP 29-320	CP 78-2114	CPCL 02-1295	L 99-233
CP 33-243	CP 79-318	CPCL 02-6848	LCP 82-89
CP 33-310	CP 80-1557	CPCL 05-1102	LHo 83-153
CP 33-425	CP 807	CPCL 05-1201	Louisiana purple
CP 34-120	CP 81-1384	CPCL 05-1791	Louisiana striped
CP 34-92	CP 82-1172	CPCL 97-2730	NCo 310
CP 36-13	CP 84-1591	CPCL 99-4455	Otaheite
CP 36-183	CP 85-1308	Black Cheribon (Creole)	POJ 213
CP 36-19	CP 85-1382	D-74	POJ 234
CP 43-47	CP 85-1432	D-95	POJ 36
CP 44-101	CP 85-1491	Ho 00-961	POJ 36M
CP 47-193	CP 86-1633	Ho 02-113	

## References

[1] Salassi M. E., Deliberto M. A. (2008). Sugarcane production in Louisiana. *A.E.A.*.

[2] Roka F. M., Baucum L. E., Rice R. W., Alvarez J. (2010). Comparing costs and returns for sugarcane production on sand and muck soils of Southern Florida, 2008-2009. *Journal of the American Society of Sugar Cane Technologists*.

[3] Gravois K. (2012). History of sugarcane research in Louisiana. *Louisiana Agricultural Magazine*.

[4] Dunckelman P. H., Breaux R. D. Breeding sugarcane varieties for Louisiana with new germplasm.

[5] Bischoff K. P., Gravois K. A. (2004). The development of new sugarcane varieties at the LSU AgCenter. *American Society of Sugar Cane Technologists*.

[6] Paliatseas E., Steib R. J., Dunckelman D. H., Chilton S. J. P. The production of true seed of sugarcane in Louisiana.

[7] Abbott E. V. (1971). History of the U.S. sugar cane field station at Houma, Louisiana. *Sugar y Azúcar*.

[8] Dunckelman P. H., Legendre B. L. (1982). *Guide to Sugarcane Breeding in the Temperate Zone*.

[9] Dunckelman P. H. (1981). Advancement of new basic sugarcane breeding lines. *Proceedings New series American Society of Sugar Cane Technologists*.

[10] Mariotti J. A., Levi C. A., Dunckelman P. H., Legendre B. L. (1991). Registration of ‘TUCCP 77-42’ sugarcane. *Crop Science*.

[11] Legendre B. L., Grisham M. P., White W. H., Garrison D. D., Dufrene E. O., Miller J. D. (1994). Registration of ‘HoCP 85-845’ sugarcane. *Crop Science*.

[12] Grassl C. O., Belcher B. A. (1953). Sugarcane breeding techniques practiced at Canal Point, Florida. *Proceedings of the International Society of Sugar Cane Technologists*.

[13] Comstock J. C., Glaz B., Tai P. Y. P., Edmé S., Morris D., Gilbert R. (2004). United States department of agriculture-agricultural research service, Sugarcane Field Station at Canal Point, Florida: past, present and future: sugar cane research institutes. *International Sugar Journal*.

[14] Deren C. W. (1995). Genetic base of U.S. mainland sugarcane. *Crop Science*.

[15] Wu C. W., Zhao P. F., Xia H. M. (2014). *Modern Cross Breeding and Selection Techniques in Sugarcane*.

[16] Zhao P., Xia H., Yang K. Sugarcane yield components and sugar content in ROC22 progeny populations.

[17] Zhang R. H., He H., Zhang G. M., Liu H. B., Li Y. R. (2011). Breeding of new sugarcane variety guitang 29 with high ratoon ability. *Sugar Crops of China*.

[18] Li C. Y., Zhang Y. G., Xiao P. X. (2012). Breeding of a new sugarcane variety dezhe 03–83. *Sugarcane and Cane Sugar*.

[19] Stokes I. E., Tysdal H. M. (1962). Significant trends in genealogies of Canal Point varieties of sugar cane. *Proceedings of the International Society of Sugar Cane Technologists*.

[20] Hebert L. P. (1964). *Culture of Sugarcane for Sugar Production in Louisiana*.

[21] Glaz B., Miller J. D., Deren C. W., Tai P. Y. P., Shine J. M., Comstock J. C. (2000). Registration of ‘CP 89-2143’ sugarcane. *Crop Science*.

[22] Tew T. L., Dufrene E. O., Cobill R. M. (2011). Registration of ‘HoCP 91–552’ sugarcane. *Journal of Plant Registrations*.

[23] Glaz B., Edmé S. J., Davidson R. W. (2009). Registration of ‘CP 00-2180’ sugarcane. *Journal of Plant Registrations*.

[24] Miller J. D., Rice E. R., James N. I., Lyrene P. M. (1976). Registration of CP 68-1067 Sugarcane1 (Reg. No. 41). *Crop Science*.

[25] Dunckelman P. H., Rice E. R., Hebert L. P. (1969). Registration of C.P. 57–603 sugarcane1 (Reg. No. 9). *Crop Science*.

[26] Glaz B., Tai P. Y., Miller J. D., Dean J. L., Kang M. S., Orsenigo J. R. (1986). Registration of ‘CP 75-1082’ Sugarcane. *Crop Science*.

[27] Tai P. Y. P., Miller J. D., Glaz B., Dean J. L., Kang M. S., Orsenigo J. R. (1984). Registration of CP 75-1632 sugarcane. *Crop Science*.

[28] Tai P. Y. P., Miller J. D., Glaz B., Dean J. L., Kang M. S., Orsenigo J. R. (1988). Registration of ‘CP 77-1776’ sugarcane. *Crop Science*.

[29] Tai P. Y. P., Miller J. D., Glaz B., Deren C. W., Shine J. M. (1991). Registration of ‘CP 78-1628’ sugarcane. *Crop Science*.

[30] Deren C. W., Glaz B., Tai P. Y. P., Miller J. D., Shine J. M. (1991). Registration of ‘CP 80-1743’ sugarcane. *Crop Science*.

[31] Tai P. Y. P., Shine J. M., Glaz B., Miller J. D., Deren C. W., Comstock J. C. (1993). Registration of ‘CP 81-1384’ sugarcane. *Crop Science*.

[32] Rice R., Baucum L., Glaz B. (2012). Sugarcane variety census: Florida 2012. *Sugar Journal*.

[33] Deren C. W., Glaz B., Shine J. M., Tai P. Y. P., Miller J. D., Comstock J. C. (1995). Registration of ‘CP 86-1633’ sugarcane. *Crop Science*.

[34] Tai P. Y. P., Shine J. M., Deren C. W., Glaz B., Miller J. D., Comstock J. C. (1997). Registration of ‘CP 88-1762’ sugarcane. *Crop Science*.

[35] Comstock J. C., Glaz B., Edme S. J. (2005). Registration of ‘CP 97-1944’ sugarcane. *Crop Science*.

[36] Glynn N. C., Zhao D., Comstock J. C. (2013). Registration of ‘CPCL 02-0926’ sugarcane. *Journal of Plant Registrations*.

[37] Rice R., Baucum L., Glaz B. (2011). Sugarcane variety census: Florida 2010. *Sugar Journal*.

[38] Rice E. R., Miller J. D., James N. I., Dean J. L. (1978). Registration of CP 70-1133 sugarcane1 (Reg. No. 45). *Crop Science*.

[39] Hebert L. P., Rice E. R., Grass C. O. (1969). Registration of C.P. 56-63 sugarcane1 (reg. no. 8). *Crop Science*.

[40] Glaz B., Tai P. Y. P., Miller J. D., Orsenigo J. R. (1990). Registration of ‘CP 80-1827’ sugarcane. *Crop Science*.

[41] Tai P. Y. P., Shine J. M., Glaz B., Deren C. W., Miller J. D., Comstock J. C. (1991). Registration of ‘CP 82-1592’ sugarcane. *Crop Science*.

[42] Glaz B., Shine J. M., Deren C. W., Tai P. Y. P., Miller J. D., Comstock J. C. (1994). Registration of ‘CP 84-1198’ sugarcane. *Crop Science*.

[43] Glaz B. (1998). Sugarcane variety census: Florida. *Sugar y Azucar*.

[44] Tai P. Y. P., Glaz B., Miller J. D., Shine J. M., Follis J. E., Comstock J. C. (2000). Registration of ‘CP 89-1509’ sugarcane. *Crop Science*.

[45] Miller J. D., Tai P. Y. P., Glaz B., Follis J. E., Comstock J. C. (2001). Registration of ‘CP 92-1641’ sugarcane. *Crop Science*.

[46] Glaz B., Follis J. E., Tai P. Y. P., Miller J. D., Comstock J. C. (2001). Registration of ‘CP 92-1666’ sugarcane. *Crop Science*.

[47] Edme S. J., Tai P. Y. P., Glaz B. (2005). Registration of ‘CP 96-1252’ sugarcane. *Crop Science*.

[48] Glaz B., Edmé S. J., Davidson R. W. (2013). Registration of ‘CP 04–1844’ sugarcane. *Journal of Plant Registrations*.

[49] Davidson W. R., Milligan S. B., Glaz B. (2011). Registration of ‘CPCL 99-4455’ sugarcane. *Journal of Plant Registrations*.

[50] Breaux R. D., Fanguy H. P., Matherne R. J., Dunckelman P. H. (1974). Registration of CP 65-357 sugarcane1 (Reg. No. 35). *Crop Science*.

[51] Anzalone L., Paliatseas E. D., Giamalva M. J., Chilton S. J. P. (1974). Registration of L 65-69 sugarcane1 (reg. no. 38). *Crop Science*.

[52] Fanguy H. P., McCormick L. L. (1981). The Louisiana sugarcane variety census for 1980. *Sugar Bull*.

[53] Gravois K., Waguespack H. Louisiana sugarcane variety Outlook. http://www.laca1.org/Presentations/2007/Sugar-Gravois.pdf.

[54] Breaux R. D., Fanguy H. P., Garrison D. (1982). Registration of CP 73-351 sugarcane1(reg. no. 58). *Crop Science*.

[55] Fanguy H. P., Garrison D., Breaux R. D. (1983). Registration of CP 74-383 sugarcane. *Crop Science*.

[56] Garrison D. D., Breaux R. D., Fanguy H. P. (1985). Registration of CP 76-331 sugarcane. *Crop Science*.

[57] Fanguy H. P., Garrison D. D., Legendre B. L. (1989). Registration of “CP 79–318” sugarcane. *Crop Science*.

[58] Milligan S. B., Martin F. A., Bischoff K. P. (1994). Registration of ‘LCP 85-384’ sugarcane. *Crop Science*.

[59] Tew T. L., Pan Y.-B. (2010). Microsatellite (simple sequence repeat) marker-based paternity analysis of a seven-parent sugarcane polycross. *Crop Science*.

[60] Miller J. D., Tai P. Y. P., Glaz B., Dean J. L., Kang M. S. (1984). Registration of CP 72-2086 sugarcane. *Crop Science*.

[61] Srivastava R. P., Singh S. P., Singh P., Singh S. B. (2006). Artificial induction of flowering in sugarcane under sub-tropical conditions—a successful approach. *Sugar Tech*.

[62] Rejesus R. M., van Ginkel M., Smale M. (1996). Wheat breeder's perspectives of genetic diversity and germplasm use. *Wheat Special Report*.

[63] Reffay N., Jackson P. A., Aitken K. S. (2005). Characterisation of genome regions incorporated from an important wild relative into Australian sugarcane. *Molecular Breeding*.

